# Botulinum Toxin in Restless Legs Syndrome—A Randomized Double-Blind Placebo-Controlled Crossover Study

**DOI:** 10.3390/toxins10100401

**Published:** 2018-09-29

**Authors:** Shivam Om Mittal, Duarte Machado, Diana Richardson, Divyanshu Dubey, Bahman Jabbari

**Affiliations:** 1 Department of Neurology, Yale University School of Medicine, New Haven, CT 06510, USA; duarte.machado@hhchealth.org (D.M.); diana.richardson@va.gov (D.R.); bahman.jabbari@yale.edu (B.J.); 2 Department of Neurology, Columbia Asia Hospitals, Sarjapur Rd, Bangalore 560102, India; 3 Department of Neurology, Mayo Clinic, Rochester, MN 55905, USA; dubey.divyanshu@mayo.edu; 4 Department of Neurology, Hartford Healthcare Ayer Neuroscience Institute, Hartford, CT 06066, USA

**Keywords:** incobotulinumtoxin A, restless legs syndrome, botulinum toxin, clinical trial, sleep, movement disorder

## Abstract

Background: Restless Legs Syndrome (RLS) is a common movement disorder with an estimated prevalence of up to 12%. Previous small studies with onabotulinumtoxin A (OnaA) for RLS have shown inconsistent results. Methods: Twenty-four patients with an International RLS score (IRLS) of >11 (moderate-severe) were enrolled in this blinded, placebo-controlled crossover study. Twenty-one patients completed the evaluations at 4, 6, and 8 weeks after each injection. One-hundred units of Incobotulinumtoxin A (IncoA) or normal saline were injected into tibialis anterior, gastrocnemius, and biceps femoris muscles each side. Results: Improvement from a severe (IRLS >21) to a mild/moderate (IRLS ≤20) score was significant at four weeks (*p* = 0.0036) and six weeks (*p* = 0.0325) following IncoA administration compared to placebo. Additionally, there was significant improvement in pain score at six weeks as measured by Visual Analogue Scale (*p* = 0.04) and the Johns Hopkins Quality of Life Questionnaire (*p* = 0.01) in the IncoA group. Definite or marked improvement on Patient Global Impression of Change was seen in 7 out of 21 patients in the IncoA group vs. 1 out of 21 patients in the placebo group at 4 weeks (*p* = 0.012). Conclusion: IncoA injection lead to a reduction in severity of RLS symptoms, pain score, and quality of life, without any adverse effects.

## 1. Introduction

Restless legs syndrome (RLS) is a sensorimotor neurological disease, characterized by an urge to move that is usually associated with abnormal sensations in the legs, symptoms that are engendered or worsened by rest, relieved by movement, and are most severe at night. The estimated prevalence is 5–12%, and it is more commonly seen in females [1,2,3]. The endothelium in the microvessels of the blood brain barrier serves as the reservoir of iron in the brain and its deficiency is linked to the pathophysiology of RLS [4]. There is a low level of iron in the cerebrospinal fluid in RLS patients compared to normal controls [5]. RLS patients, particularly those with more severe RLS, report significant deficits in physical functioning, role functioning, mental health, general health, and vitality, as well as increase in bodily pain compared with the general population [6]. The first line treatment for RLS is dopaminergic medications such as dopamine agonists and α-2-δ calcium-channel ligands [7,8]. Long-term treatment with dopaminergic therapy poses a risk of worsening of symptom severity called augmentation, and other side effects such as leg edema and behavior changes. Even with the regular treatment, RLS symptoms remained unchanged or worsened in 45% of the patients [9]. Thus, there is a need for a treatment for patients with refractory RLS which has a safe side-effect profile and tolerability.

Botulinum neurotoxins (BoNTs) are the most potent toxins, and are produced by various strains of clostridium botulinum bacteria. Serologically, seven types of botulinum toxins have been identified (A, B, C, D, E, F, G) from which, only types A and B are available in clinical practice. More recently, several subtypes have been recognized for each type of the toxin (for example, subtypes A1, A2, A3). Currently, more than 40 unique BoNTs have been identified, which display heterogeneity ranging from very little (<1%) to higher than 35% [10]. New data indicate that hybrid toxins exists in nature and botulinum toxin-like molecules exist in several non-clostridial bacteria such as weissella and enterococcus [11]. The molecular structure of botulinum toxin consists of a heavy chain (100 KD) and a light chain (50 KD), held together by a disulfide bond. After reaching the nerve endings, BoNTs first use a polysialoganglioside receptor represented in the surface of the nerve membrane. Then it passes through a second transmembrane receptor for which type A toxin is from the SV2 family of proteins and type B toxin is luminal domain of synaptotagmin [11]. Translocation of the toxin from inside of the synaptic vesicle into the cytosol is triggered by the acid environment inside the vesicle. In the cystosol, there is a reduction in the disulfide bond freeing the active part of the toxin i.e., the light chain (L) moiety of the toxin. Recent literature indicates that this L reduction and refolding is managed by a chaperone-redox machinery which includes the Thioredoxin–Thioredoxin Reductase system working with a cytosolic chaperone protein called HSP90 [12]. Therefore, the light chain is a protease that attaches itself to synaptic SNARE (Soluble NSF Attachment protein Receptor) proteins and deactivates them to prevent vesicular rupture and release of neurotransmitters. There are different SNARE proteins working at the synapse such as SNAP25 and synaptobrevin. Type A toxin and type B toxin target the SNAP25 and synaptobrevin, respectively. Recently, several SNAP proteins designated different numbers have been identified which are the target of different toxin subtypes. The commercially available BoNTs in the U.S. are type A (Onabotulinumtoxin A or Botox, Incobotulinumtoxin A or Xeomin, and Abobotulinumtoxin A or Dysport) and type B (Rimabotulinumtoxin B or Myobloc).

BoNTs inhibit the release of acetylcholine and other neurotransmitters by blocking the SNARE proteins which are necessary for vesicle exocytosis and the release of neurotransmitters. This causes a sustained blockage of release of acetylcholine in the neuromuscular junction, leading to chemodenervation. This is the primary reason it helps in conditions such as spasticity, cervical dystonia, blepharospasm, hemifacial spasm, and focal dystonias. BoNTs also alleviate neuropathic pain in animals through several mechanisms: blocking the release of pain mediators (glutamate, substance P, calcitonin gene related peptide (CRGP)) from peripheral terminals, dorsal root ganglia (DRG), and spinal cord neurons; decreasing local inflammation around nerve terminals; deactivation of sodium channels and decreasing sympathetic transmission [13]. Thus, there is an expanding literature on the role of BoNTs in pain conditions such as chronic migraine, post herpetic neuralgia, post-traumatic neuralgia, trigeminal neuralgia, and other neuropathic pain conditions.

Recent literature suggests that the pathophysiology of restless legs syndrome includes impaired cortical sensory-motor integration [14]. Additionally, there is physiological evidence for hyperexcitability of the cortical-striatal-thalamic-cortical network in RLS leading to hyperexcitability of spinal motor neurons [15]. Injection of BoNTs into the muscles or skin significantly reduces the increased sensitivity of motor and sensory systems. Thus, intramuscular injection of BoNTs, in addition to muscle relaxation causing inhibition of acetylcholine, significantly reduces the discharge of muscle spindles [16]. Muscle spindles report the length and tension of the muscle to the spinal cord via their major excitatory input. Furthermore, there is evidence that intramuscularly or intradermally injected botulinum toxins can reach the spinal cord from the site of injection (via retrograde/anterograde transmission) and directly influence the spinal cord neurons [17].

Three open label studies and a single, small, randomized double-blind pilot study have evaluated the efficacy of BoNTs in RLS [18,19,20,21]. These studies demonstrated mixed results with an unclear consensus. There is evidence that BoNTs may modulate afferent sensory fiber firing and thereby reduce the central sensitization and pain perception [13]. To date, the present trial is the largest double-blinded placebo-controlled study assessing the efficacy and safety of botulinum neurotoxin type A, namely incobotulinumtoxinA (IncoA) in refractory RLS patients.

## 2. Results

**Figure 1:**
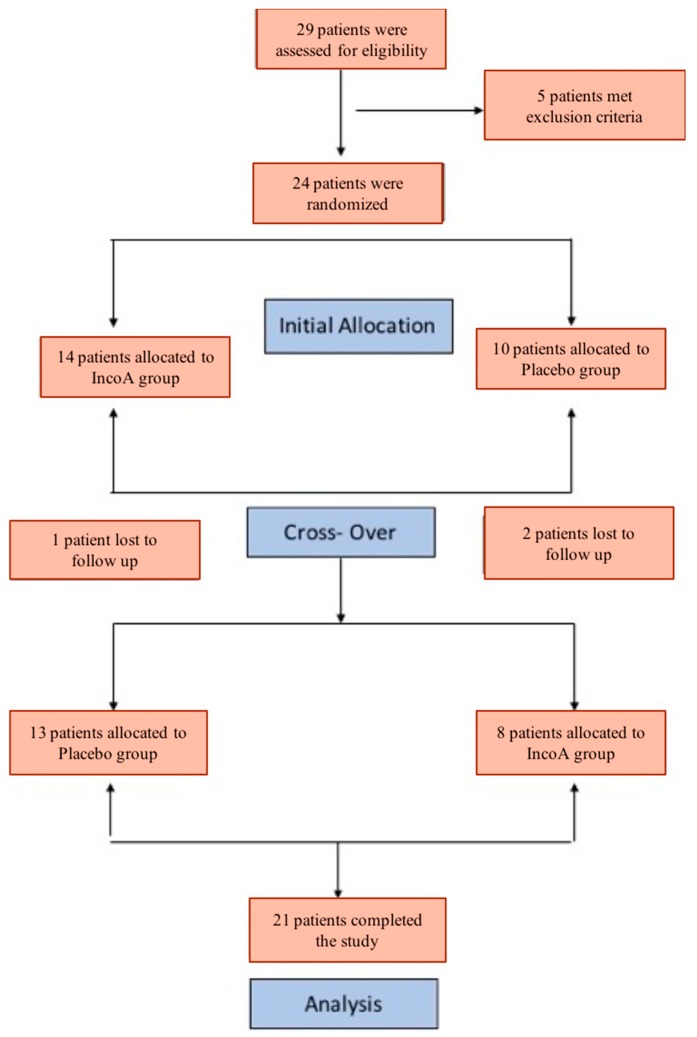
CONSORT Diagram.

A total of 29 subjects were screened and 24 patients completed enrollment in the study (Figure 1—Consort diagram). Twenty-one subjects completed the study. Table 1 summarizes the baseline characteristics for each sequence group. The mean age of the patients was 61 years (range: 37–92 years) with 11 female patients in the study. At baseline, all patients had moderate (1 patient), severe (13 patients), or very severe (7 patients) symptoms on the IRLS rating. This was not statistically different between the two study groups. The rest of the parameters, such as the John Hopkins Quality of Life Questionnaire (QoL), the sleep score from Medical Outcome Study (MOS), the Visual Analog Scale for pain (VAS), and the Epworth Sleepiness Scale (ESS), did not show any statistical difference between the study groups at the baseline visit. (Table 1).

There was significant improvement from a severe (IRLS > 21) to a mild/moderate (IRLS ≤ 20) score at 4 weeks (*p* = 0.0036) and 6 weeks (*p* = 0.0325) following IncoA administration compared to placebo. No significant improvement was detected at 8 weeks (*p* = 0.0670). Additionally, there was significant improvement in pain score by VAS at 4 weeks (*p* = 0.01) and in the Johns Hopkins QoL questionnaire at 6 weeks (*p* = 0.04) in the IncoA group. Definite or marked improvement on patient global impression of change was seen in 7 out of 21 patients in the IncoA group vs. 1 out of 21 patients in the placebo group at 4 weeks (*p* = 0.012). The ESS and the sleep questionnaire from MOS were not significantly different between study and placebo groups (Table 2). There was no weakness noted in either IncoA or placebo groups at 6 weeks post-injection on muscle power testing using Medical Research Council (MRC) grading.

## 3. Discussion

Our study is the largest double-blinded placebo-controlled study for the use of BoNTs in RLS to date. In our crossover study, IncoA injection in leg muscles using electromyographic (EMG) guidance (anterior tibialis, biceps femoris, and gastrocnemius) significantly improved the IRLS scores at 4 and 6 weeks in patients with severe RLS (IRLS > 21). Improvement of Quality of life (QoL), and pain and discomfort (measured with VAS) were statistically significant only at 6 and 4 weeks, respectively. The lack of statistical significance at 8 or 12 weeks in this study in our view represents two issues. Firstly, the efficacy of BoNT therapy in a clinical condition is highly dose dependent. It is possible that a larger dose could have lengthened the duration of response. Secondly, the study was underpowered as a result of the small number of patients. There is a trend towards improvement in quality of life in the IncoA group at both 4 and 8 weeks (Table 2). With a larger cohort, these values would have reached statistical significance beyond six weeks.

For the last two decades, BoNTs have had wide therapeutic implications in the treatment of several neurological conditions such as movement disorders including dystonia, hemifacial spasm, tremor, and tic disorder; post-stroke spasticity; autonomic dysfunction such as sialorrhea and hyperhidrosis; and several pain conditions such as chronic migraine, and neuropathic pain. Overall BoNT has a good safety profile with dose related side effects [13,22,23,24,25].

Previous studies assessing the efficacy of botulinum toxins in RLS produced conflicting results. In 2006, Rotenberg [21] first reported improvement of urge to move legs, nocturnal restlessness, and leg discomfort after injection of Onabotulinumtoxin A (OnaA) into tibialis anterior muscles. In an open label study, eight patients with moderate to severe RLS were injected with OnaA (25 units in each tibialis anterior muscle) [18]. The authors found statistically significant improvement in IRLS score, pain, and patient global impression of change at 4 weeks after IncoA injection. Ghorayeb [20], in another open label study, assessed the efficacy of intradermal injection of AbobotulinumtoxinA (Abo-A) (250 units to the anterior and posterior thigh muscles) in 26 patients with severe RLS. All patients had improvement in IRLS score; reduction of IRLS score was noted in 8% and 19% of the patients at 4 and 6 weeks, respectively. The authors considered the results negative as only 6 patients (out of the 9 expected) in the first phase of the study (week 2) showed >50% improvement in IRLS score. Nahab [19], conducted a double-blind placebo-controlled study in six patient from whom three were injected with OnaA and three were injected with placebo. The OnaA dose was 90 units injected under EMG guidance into each leg muscle (quadriceps femoris: 40 units in four sites; tibialis anterior: 20 units in two sites; gastrocnemius: 20 units in two sites; soleus: 10 units in one site). There was no significant improvement of the IRLS score or clinical global improvement score in the OnaA group compared to placebo.

It is worthwhile to discuss the technical differences between our study and previously negative studies, attempting to explain the difference between our results and theirs. In the study of Ghorayeb [20], the authors used intradermal, grid-like injections. This technique, which has shown promise in neuropathic pain, may not work for RLS since it does not significantly influence the major and active muscles involved in RLS during evening and nocturnal movements (tibialis anterior and gastrocnemius). As for the study of Nahab [19], the dose of the toxin that we used in the tibialis anterior and gastrocnemius muscles might have been the main factor. Our dose for each of these muscles was twice as large as that used in their study (40 versus 20 units per muscle). Although the toxin doses are not interchangeable, most comparative clinical trials use a 1:1 ratio between the OnaA and IncoA toxins. It is also possible that OnaA and IncoA in certain disease conditions may affect the sensory-motor systems differently.

Our study has limitations. The relatively small size of the studied cohort might have underpowered some of statistical assessments. Inclusion of polysomnographic assessments could have provided valuable information and objective data on sleep quality in RLS patients. As neither of the two reported blind studies (ours and that of Nahab et al. [19]) reported any significant muscle weakness with the applied doses, future studies may consider injecting slightly higher doses of BoNT into the leg muscles to evaluate whether even longer relief of symptoms can be achieved.

## 4. Conclusions

The results of our current randomized, double-blind, placebo-controlled, crossover trial are encouraging. This study showed that IncoA at the applied dose can reduce the severity of symptoms in RLS at six weeks. Moreover, the quality of life of the RLS patient can be improved for up to six weeks post-injection, and pain and discomfort diminishes for four weeks.

## 5. Materials and Methods

The Yale Institutional Review Board approved the study protocol on 6 September 2012. The informed written consent was obtained from all study participants. The study was registered under ClinicalTrials.gov (NCT01931878). The study was conducted at the Yale Movement Disorder Center in New Haven, Connecticut, US. The study was designed as a randomized, double-blind, placebo-controlled crossover study. The crossover happened at the end of 12 weeks following the first injection of IncoA or saline (Figure 1).

The patients were injected using a fixed dose approach to the anterior tibialis, biceps femoris, and gastrocnemius muscles bilaterally. The dose per side was 40 units, 40 units, and 20 units for tibialis anterior, gastrocnemius, and biceps femoris muscles, respectively. For tibialis anterior, the dose was divided into two points. For the gastrocnemius muscles, 20 units each was injected into the medial and lateral gastrocnemius. The total dose per side and per session of injection (bilateral) was 100 and 200 units, respectively. The dilution used was 1:1 with 100 units of IncoA mixed with 1 mL of preservative-free saline. A similar injection technique was used in the placebo group using an identical amount of normal saline. The injection syringes with identical volumes were prepared by a nurse and thus was blind to the injectors/investigators. A 27-gauge sterile needle was used for the injections administered by movement disorders neurologists under EMG guidance.

Patients were assessed for any new symptoms or side-effects during the study follow up visits and telephone interviews. Patients had a neurological exam with the Medical Research Council (MRC) scale for motor power testing of leg muscles at baseline and 6 weeks post injection in order to document any weakness.

### 5.1. Outcome Measures

Clinical assessments were performed at the beginning, four weeks, six weeks, and eight weeks after the respective injections. Severity of RLS symptoms was assessed by International RLS score. Visual Analog Scale (VAS 0–10) and Johns Hopkins Quality of Life questionnaire (QoL) were utilized to assess pain perception and quality of life, respectively. Sleep disturbances were assessed by Epworth Sleepiness Scale (ESS) and Sleep Scale from Medical Outcome Study (MOS). The patient’s self-estimated clinical improvement was based on the patient global impression of change (PGIC, range 0–7). A rater concealed from the treatment allocation performed the clinical assessments. Any adverse events were recorded at each visit and during a telephone conversation.

The primary endpoint was the change in IRLS score from the baseline at 6 weeks. Secondary outcomes were change in quality of life (QoL), sleep disturbances (ESS and sleep scale of MOS scale), pain assessment (VAS), and patient’s perception of change (PGIC). MOS is a 12-item self-report sleep measure designed to provide a concise assessment of important dimensions of sleep, including initiation, maintenance, respiratory problems, quantity, perceived adequacy, and somnolence [26]. The differences in the two groups (IncoA or placebo) were determined by calculating the difference in the IRLS score, VAS, QoL, MOS, and PGIC score before and after the injection at 4, 6, and 8 weeks.

### 5.2. Study Population

All study subjects had a clinical diagnosis of RLS meeting the criteria set forward by the International RLS Study Group in 2012 [3]. Enrolled patients failed at least two drugs for treatment of RLS, and had an International Restless Legs Syndrome (IRLS) Score of above 11 (moderate to severe RLS). During the study period, pharmacological and/or non-pharmacological treatments for RLS were not modified.

Subjects aged less than 18 years, with sensitivity or allergy to BoNTs, presence of neuromuscular junction disorders, history of BoNT injections in the past 4 months, anesthetic medications within two weeks and corticosteroid injections in the past 1 month, pregnancy/breastfeeding, and pre-existing significant medical conditions (i.e., cardiovascular, neoplastic, infectious, or autoimmune disorders), and those with swallowing or breathing difficulties, were excluded from the study. Subjects were not to participate in another investigational study during the course of this study.

### 5.3. Randomization and Blinding

Randomization of subjects was performed using computer-generated software. IncoA or placebo was dispensed by a nurse who was not involved in the patient interview or rating. All data were stored in a password protected computer. The study was performed in a blinded fashion whereby all study participants, study investigators, and caregivers were blinded. Merz Pharmaceuticals GmbH (Frankfurt am Main, Germany) provided IncoA for the study.

The primary objective for this double-blind, placebo-controlled, crossover trial was to assess change in the IRLS score at 6 weeks. Twenty-four subjects with moderate to severe RLS symptoms were randomized to receive IncoA during the first phase of the study (12 weeks) and placebo (saline) during the second phase (12 weeks) of the study or placebo during the first phase of the study and IncoA during the second phase of the study (see Figure 1). For each study participant, the change in IRLS score from baseline (time of injection) to 4, 6, and 8 weeks after the injections was calculated and recorded.

Fisher’s exact test was used to test the null hypothesis i.e., there is no difference in IRLS score from the baseline visit (time of injection) to 6 weeks after the injection (between IncoA and placebo study groups). For the secondary end points, Wilcoxon signed rank test was utilized. A non-parametric Levene’s test was used to verify the equality of variances in the samples (homogeneity of variance) (*p* > 0.05). IBM SPSS Statistics for Windows version 23 (IBM Corp., Armonk, NY, USA) was used to perform all of the statistical analyses, and a *p*-value lower than 0.05 was used to denote statistical significance.

The calculation of sample size was based on our preliminary observations of 70% efficacy in the experimental group versus 25% anticipated as the placebo effect. (Power = 80% and significance level alpha of 5% (sealed envelope sample size calculator)).

## Figures and Tables

**Table 1:** Baseline Characteristics.

Baseline Characteristics	Placebo (*n* = 10)	Standard Deviation	Botulinum Toxin (*n* = 14)	Standard Deviation	*p* Value
Age, years (range) *	60.5 (44–92)	14.75	64 (37–78)	13.49	0.94
Female (%) **	5 (62.5)	-	6 (46.2)	-	0.66
IRLS (range) *	30.5 (25–38)	4.66	27 (11–37)	7.13	0.21
QOL (range) *	34 (26–61)	11.06	31 (18–44)	7.93	0.24
MOS (range) *	35.5 (27–43)	4.99	38 (27–48)	6.21	0.34
VAS (range) *	5.5 (1–9)	3.07	5 (0–10)	3.07	0.27
ESS (range) *	13.5 (3–23)	7.18	10 (1–21)	6.29	0.41

IRLS—International Restless Legs Syndrome (RLS) score, VAS—Visual Analog Scale, QOL—Johns Hopkins Quality of Life questionnaire, ESS—Epworth Sleepiness Scale, MOS—Sleep Scale from Medical Outcome Study. * Mann–Whitney U test; ** Fisher exact test.

**Table 2:** Results.

Variables		Placebo (*n* = 24)	Standard Deviation	Botulinum Toxin (*n* = 24)	Standard Deviation	*p* Value *
**QOL**	4 weeks (range)	30.5 (15–42))	7.35	36 (16–48)	0.06	9.52
	6 weeks (range)	32 (14–42)	9.22	36 (11–48)	**0.04**	9.55
	8 weeks (range)	32 (14–42)	8.69	36 (11–49)	0.08	9.88
**VAS**	4 weeks (range)	6 (1–10)	2.30	3 (0–8)	**0.01**	2.74
	6 weeks (range)	4 (1–10)	2.63	4 (0–10)	0.09	2.82
	8 weeks (range)	4 (1–10)	2.61	3 (0–8)	0.09	2.73
**ESS**	4 weeks (range)	8 (1–23)	7.12	9 (1–21)	0.73	6.36
	6 weeks (range)	9 (1–22)	6.64	10 (1–22)	0.46	6.29
	8 weeks (range)	9 (1–33)	7.97	10 (1–22)	0.22	6.48

VAS—Visual Analog Scale, QOL—Johns Hopkins Quality of Life questionnaire, ESS—Epworth Sleepiness Scale. * Wilcoxon signed rank test.
